# Perceive–Assess–Dose–Safeguard: a safety-gated state–action grammar for psychotherapy micro-decisions in computational psychiatry

**DOI:** 10.3389/fpsyt.2026.1749364

**Published:** 2026-03-13

**Authors:** Eik Niederlohmann

**Affiliations:** Department of Psychosomatic Medicine and Psychotherapy, Kliniken Erlabrunn, Breitenbrunn, Germany

**Keywords:** active inference, computational psychiatry, computational psychotherapy, human-in-the-loop, interpretable AI, Mini-ICF-APP, psychotherapy process coding, SPICE

## Abstract

Psychotherapy unfolds as a sequence of rapid micro-decisions under uncertainty. Within seconds, clinicians integrate verbal, paraverbal, embodied, and relational cues, estimate the patient’s momentary capacity for affective work, choose an intervention dose, and apply stop rules to prevent overwhelm and rupture. Computational psychiatry offers principled frameworks for sequential decision-making, but progress in computational psychotherapy remains constrained by the lack of clinically grounded, machine-readable grammars that capture therapist micro-decisions in context. I introduce the Perceive-Assess-Dose-Safeguard (PAD-S) decision matrix as a safety-gated state–action grammar for psychotherapy micro-decisions. PAD-S formalizes four “front-of-system” signals—defensive/avoidant organization (DEF), anxiety/arousal and tolerance (ANX), patient progression toward direct experience and action (PRO), and self-attack/shame processes (SUP)—together with three safety thresholds (A–C) that gate intervention dose. Each decision point can be logged as an “episode line” (trigger, state, threshold, action, and expected functional impact), enabling transcript annotation and structured datasets. PAD-S is grounded in experiential dynamic psychotherapy (EDT/ISTDP) yet expressed as an orientation-translatable representation layer: DEF can be read as avoidance/safety behavior, ANX as arousal/tolerance, PRO as approach and value-consistent action, and SUP as self-criticism/shame. I show how PAD-S trajectories can interface with hybrid neural–cognitive models such as SPICE to discover sparse, interpretable equations of process change, and I outline testable hypotheses and feasible pilot studies (reliability, outcome linkage, and modeling) to evaluate the framework. computational psychiatry; computational psychotherapy; psychotherapy process coding; interpretable AI; human-in-the-loop; active inference; SPICE; Mini-ICF-APP

## 1 Introduction

Computational psychiatry has advanced mechanistic accounts of learning and decision-making, and related work on computational psychological therapy has argued that formal models can inform psychotherapy training and personalization (1–3). Yet psychotherapy is not a laboratory task: it unfolds in time, in context, and within a relationship, often under strong affect and uncertainty—precisely the conditions that computational psychiatry has identified as essential to model explicitly (4).

A central bottleneck for computational psychotherapy is representational. Many computational approaches can learn from sequential data, but psychotherapy transcripts rarely come with clinically meaningful, machine-readable labels of the therapist’s moment-to-moment decisions. A recent scoping review of computational methods in psychotherapy highlights substantial heterogeneity in how process is operationalized, coded, and linked to outcomes (5). In parallel, large language models are increasingly used for psychotherapy-related tasks, which raises both opportunities and governance challenges; however, their utility still depends on clear target representations and high-quality supervision signals (6).

Hybrid neural–cognitive approaches can mitigate the interpretability gap by combining flexible sequence models with equation discovery. For example, SPICE automates the discovery of sparse and interpretable cognitive equations from sequential behavior (7). Such approaches have also been discussed in the broader context of automating parts of the scientific workflow, including hypothesis generation and model selection (8). To bring these tools to psychotherapy, we need a compact state–action grammar that is clinically grounded, safety-aware, and feasible to annotate.

The Perceive-Assess-Dose-Safeguard (PAD-S) decision matrix is proposed as such a grammar. PAD-S is theoretically grounded in experiential dynamic psychotherapy traditions (EDT/ISTDP), which emphasize real-time monitoring of defensive organization, anxiety/arousal tolerance, patient progression, and self-attack/shame processes to titrate the dose of affective work and protect the therapeutic relationship (9–12). PAD-S grew out of an earlier transdiagnostic “Conflict Square Algorithm” that aimed to support functional formulation and documentation (13), but it is refined here as an explicit state–action representation for transcript annotation and computational modeling.

Importantly, PAD-S is not presented as “school-neutral” in the sense of being theory-free. Instead, it is an orientation-translatable intermediate representation: its state variables can be mapped to constructs familiar in multiple traditions (e.g., DEF as avoidance or safety behavior; ANX as arousal and tolerance; PRO as approach, emotional access, and value-consistent action; SUP as self-criticism and shame). This translation stance is intended to make PAD-S usable across modalities without requiring endorsement of psychodynamic metatheory, while still preserving the clinically pragmatic heuristics that motivated the model.

PAD-S is also designed to complement—not replace—existing psychotherapy process measurement traditions. Technique-specific coding systems (e.g., motivational interviewing skill codes, CBT adherence/competence scales), Q-set approaches (e.g., psychotherapy process Q-set), and relational theme methods (e.g., core conflictual relationship themes) capture important dimensions of process. PAD-S adds a safety-gated decision layer that explicitly represents the therapist’s momentary assessment of tolerance and the consequent dose/stop-rule choices, which can be integrated with richer descriptive systems when needed (5).

**Figure 1:**
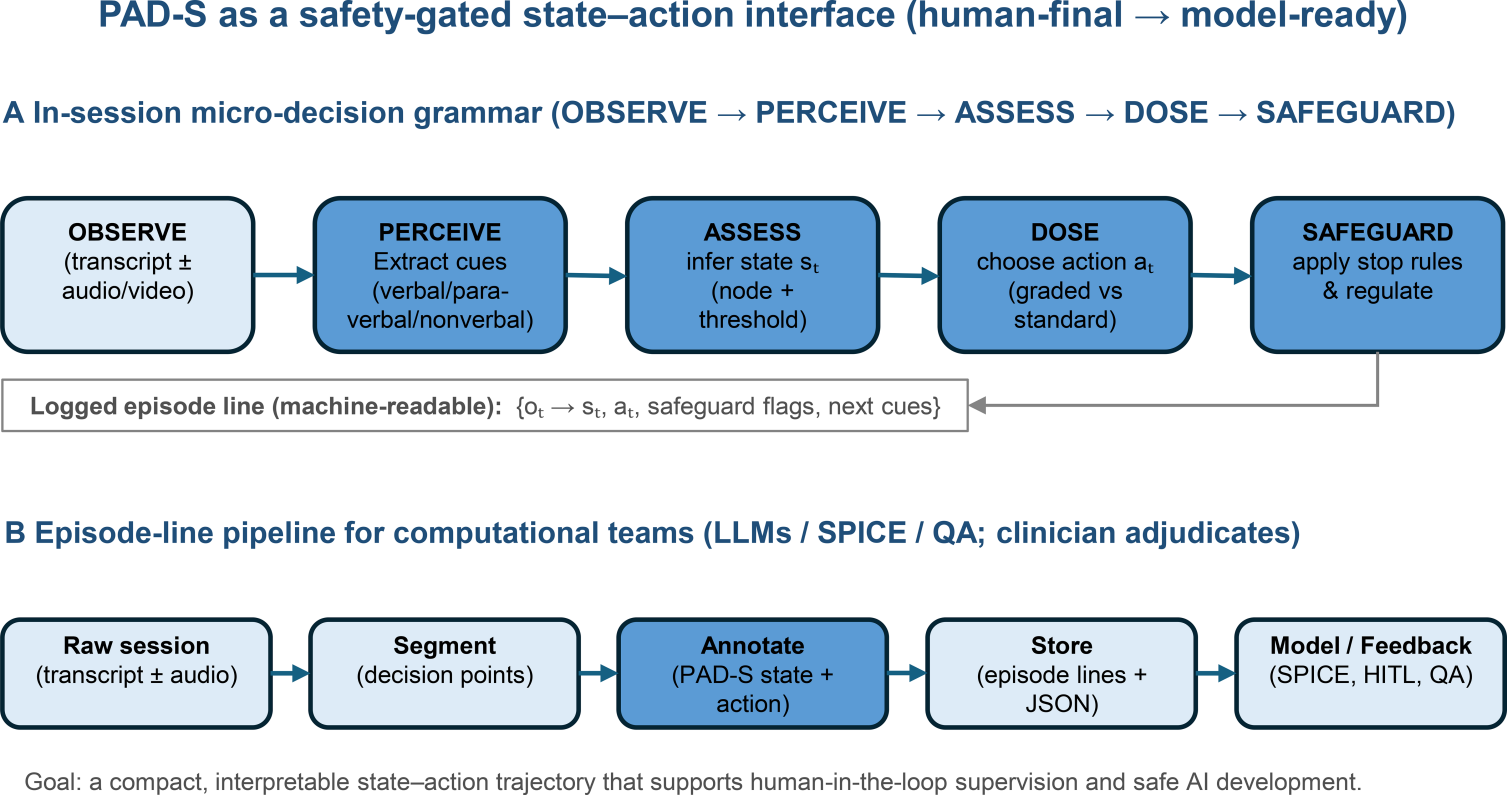
PAD-S as a safety-gated state–action interface (human-final → model-ready). **(A)** In-session micro-decision grammar. PAD-S is represented as a safety-gated loop: OBSERVE (transcript ± audio/video) → PERCEIVE clinically actionable cues (verbal/paraverbal/nonverbal) → ASSESS the current interaction state s_t_ (front-of-system node + tolerance threshold) → DOSE the next-step intervention/action a_t_ (graded vs. standard) → SAFEGUARD by applying explicit stop rules and regulation when overload/shame-collapse markers emerge. Each decision point is logged as a machine-readable episode line (e.g., o_t_ → s_t_, a_t_, safeguard flags, next cues). **(B)** Episode-line pipeline for computational teams. Raw sessions are segmented into clinically meaningful decision points, annotated with PAD-S state + action labels, stored as episode lines (e.g., JSON), and converted into state–action trajectories for downstream modeling (e.g., interpretable hybrid approaches) and human-in-the-loop feedback/quality assurance, while keeping the clinician in human-final control.

**Figure 1** provides an overview of PAD-S as (i) a clinical micro-decision workflow and (ii) a data pipeline that converts transcript episodes into structured state–action trajectories amenable to downstream modeling. **Box 1** provides a minimal formalization framing PAD-S as a safety-constrained policy over discrete states.

## 2 The PAD-S decision matrix

**Figure 2:**
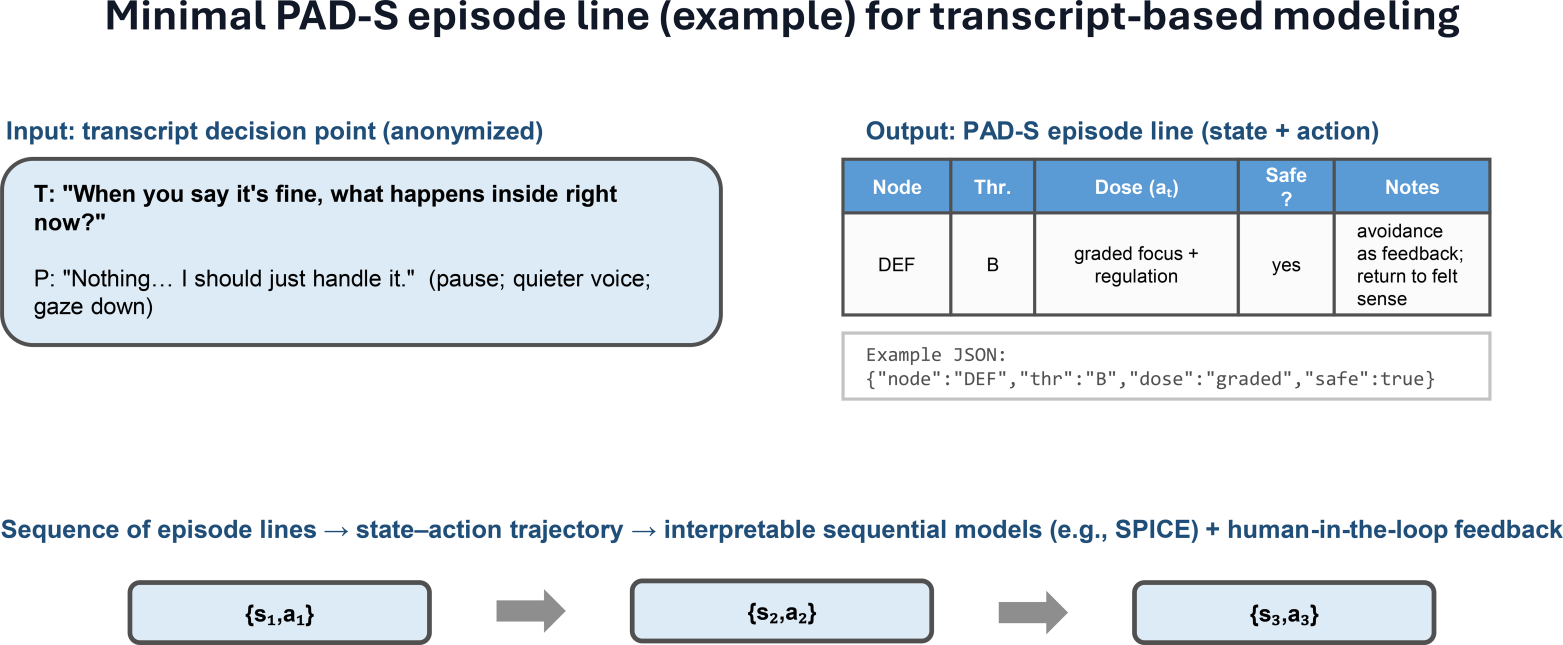
Minimal PAD-S episode line (example) for transcript-based modeling. A single transcript decision point (T = therapist; P = patient; anonymized illustrative example) is mapped to a compact PAD-S episode line capturing the assessed Node (front-of-system signal), Thr. (tolerance threshold), selected intervention Dose/Action (a_t_; graded vs. standard), a Safeguard indicator (whether stop-rule/regulation was applied), and brief notes. The episode line can be stored as a structured record (e.g., JSON) and concatenated across decision points to form state–action trajectories {(s_1_,a_1_), (s_2_,a_2_), …}, enabling interpretable sequential modeling and human-in-the-loop supervision/feedback.

PAD-S is a compact decision matrix that represents psychotherapy micro-decisions as a sequence of safety-gated state–action steps. At each decision point, the therapist (i) perceives clinically relevant cues, (ii) assesses the current “front-of-system” state and tolerance threshold, (iii) selects an intervention dose and type, and (iv) safeguards the process with explicit stop rules when risk markers indicate overload or shame-related rupture risk (**Figure 1**). **Figure 2** illustrates a minimal PAD-S episode line representation for transcript-based modeling.

### 2.1 Perceive: cues that matter for micro-decisions

Perception in PAD-S refers to identifying momentary cues that are actionable for process management. These cues can be verbal (content, contradictions, commitment language), paraverbal (tempo, prosody), embodied (tension patterns, breath, gaze), and relational (alliance signals, ruptures, responsiveness). PAD-S does not assume that any single channel is privileged; rather, cues serve as proxies for the current state of affect regulation and action readiness that inform dosing and safeguarding.

### 2.2 Assess: four front-of-system nodes and three tolerance thresholds

The core state representation in PAD-S combines a categorical node (DEF, ANX, PRO, SUP) with a tolerance threshold (A, B, C). Nodes summarize what dominates the process in the moment; thresholds gate how much affective “pressure” or exposure can be safely applied without pushing the system into overload.

Nodes (front-of-system signals): DEF captures defensive or avoidant organization (e.g., detours, intellectualization, reassurance seeking, disengagement). ANX captures anxiety/arousal and tolerance (e.g., somatic tension, dysregulated arousal, cognitive-perceptual disruption). PRO captures progression: direct access to feeling, clarity of wish/goal, and readiness for value-consistent action. SUP captures self-attack/shame processes (e.g., self-criticism, moralizing collapse, joy-to-attack switches). While these labels are grounded in EDT/ISTDP, they can be translated into common constructs used across modalities (avoidance, arousal tolerance, approach/action, self-criticism).

Thresholds (A-C): Threshold A indicates a regulated window in which standard interventions are typically tolerated. Threshold B indicates heightened arousal or fragility requiring graded dosing, shorter exposures, and more frequent regulation checks. Threshold C indicates overload markers (e.g., cognitive-perceptual disruption, panic-level dysregulation, or shame collapse), requiring immediate safeguarding (stop deepening, regulate, protect positives, repair alliance) before any further exposure or pressure is attempted.

### 2.3 Dose and safeguard: action selection under safety constraints

Dose refers to the intensity and format of the next-step intervention (e.g., standard vs. graded), as well as the choice of node-appropriate actions (e.g., brief defense-blocking vs. supportive reflection; affect focus vs. grounding; protect positives vs. interpretation). Safeguard refers to explicit stop rules that prioritize safety and alliance: if C-level markers appear, the policy requires shifting from deepening to regulation and repair.

This safety-gated design is intended to make the model usable for computational purposes without implying automation of clinical decisions. PAD-S is a descriptive and prescriptive grammar for human decision-making: it makes the clinician’s implicit gating logic explicit and recordable.

### 2.4 The episode line: a minimal unit for annotation and functional linkage

To support transcript annotation and downstream modeling, PAD-S uses the “episode line” as a minimal recording unit. An episode line captures (i) a trigger/cue, (ii) the assessed node(s), (iii) the threshold (A-C), (iv) the selected action and dose format, and (v) the expected functional impact (e.g., on planning, endurance, social interaction, self-care). This functional orientation can be linked to standardized domains such as the Mini-ICF-APP, a widely used instrument for assessing activity and participation limitations in mental disorders (14). The episode line may also include a planned re-check interval; this refers to when the specified functional target (e.g., a Mini-ICF-APP domain) should be reassessed in routine care (for example after ~4–6 weeks of weekly sessions) rather than to the timing of session-by-session coding.

**Supplementary Materials S1**-**S3** provide an implementable codebook, a decision matrix, annotated examples, a JSON schema for episode lines, and a cross-walk to functional domains. **Box 1** summarizes a minimal computational formalization of PAD-S that is sufficient to situate the framework within control-theoretic and hybrid-modeling perspectives.

Box 1 **Minimal formalization of PAD-S as a safety-gated state-action policy.**

Unit of analysis: decision point/episode t within a session.

Observations: o_t = cues perceived by the therapist (verbal, paraverbal, embodied, relational).

State: s_t = (node_t, thr_t, ctx_t), where node_t ∈ {DEF, ANX, PRO, SUP} and thr_t ∈ {A, B, C}.

Actions: a_t ∈ A, where A includes node-appropriate interventions (e.g., clarify/invite, block a defense, focus affect, regulate, protect positives, repair alliance).

Dose format: f_t ∈ {standard or graded}.

Safety constraint (stop rule): if thr_t = C, restrict actions to safeguarding and regulation until thr_t returns to A or B.

Episode line (log record): l_t = (trigger_t, s_t, a_t, f_t, expected_function_t).

Structured dataset: D = {l_t} for t = 1..T across sessions/cases.

Pseudo-code (high-level):

1) o_t ← observe()

2) node_t ← classify_front_of_system(o_t)

3) thr_t ← estimate_tolerance(o_t)

4) if thr_t == C: a_t ← safeguard_and_regulate(node_t)

else: a_t ← choose_next_step(node_t, thr_t) (standard or graded)

5) l_t ← log(trigger_t, node_t, thr_t, a_t, f_t, expected_function_t)

6) proceed to next decision point

State and observations. At each decision point t, PAD-S uses observable cues o_t (verbal, paraverbal, embodied, relational) to approximate a latent interaction state s_t = (node_t, thr_t, ctx_t), where node_t ∈ {DEF, ANX, PRO, SUP} and thr_t ∈ {A, B, C}.

Actions. The clinician selects a next-step action a_t from a small, interpretable set A (e.g., clarify_defense, regulate_ground, graded_exposure, standard_exposure, protect_positives, alliance_repair).

Policy. PAD-S defines a safety-gated policy π_PAD-S(a_t|s_t) implemented as a node × threshold lookup rule (**Supplementary Material S1**). A hard safety constraint applies: if thr_t = C (cognitive–perceptual disruption or shame collapse), deepening actions are disallowed and the action must prioritize regulation/repair (and protection of positives when relevant).

Episode line. Each decision point is recorded as e_t = (trigger_t, s_t, a_t, expected_function_t, recheck_t). The resulting trajectory {e_t} provides discrete, human-interpretable labels for downstream modeling (e.g., hybrid neural–cognitive approaches; Section 4) and for training/supervision feedback.

Minimal pseudocode:

observe cues o_t.

infer node_t, thr_t.

if thr_t == C: a_t ← regulate/repair (+ protect_positives if needed).

else: a_t ← lookup(node_t, thr_t).

log episode line e_t.

## 3 PAD-S as a control problem: resource-rational and active-inference perspectives

PAD-S can be read as a high-level control policy for a coupled human system (patient–therapist dyad) operating under uncertainty and safety constraints. At each decision point, the clinician performs approximate state estimation (node and threshold) and selects an action intended to move the process toward progression (PRO) while avoiding catastrophic states (e.g., overload or shame collapse).

From a resource-rational perspective, therapists face computational constraints: they must allocate attention, working memory, and inference to the most informative cues and actions in real time. Rational metareasoning models and resource-rational analysis provide a principled language for why therapists may prefer simple heuristics and why dosing and safeguarding functions are essential under limited cognitive control resources (15, 16). PAD-S makes these heuristics explicit and thus testable.

From an active-inference perspective, PAD-S corresponds to (i) selecting observations that reduce uncertainty (Perceive), (ii) inferring a latent state of the interaction (Assess), (iii) choosing a policy (Dose) that trades off epistemic and pragmatic value, and (iv) enforcing safety priors that prevent high-cost prediction errors (Safeguard) (17). Recent work extending active inference toward social actors and context-sensitive regulation provides a natural conceptual bridge for modeling dyadic psychotherapy as an embedded process rather than an isolated decision-maker (18).

## 4 Mapping PAD-S to hybrid neural–cognitive models such as SPICE

PAD-S produces discrete state–action trajectories that are well suited to hybrid modeling. Each episode line can be interpreted as a time step t with state s_t = (node_t, thr_t, context_t) and action a_t (**Box 1**). Sequences of episode lines across a session form trajectories that encode how therapist actions interact with patient state transitions (e.g., DEF → ANX → PRO, or PRO → SUP).

SPICE combines sequence modeling with sparse equation discovery to recover interpretable structural relationships from behavioral trajectories (7). In psychotherapy, PAD-S trajectories can serve as the observable scaffold: SPICE (or related methods) can be applied to discover compact equations that predict transitions between nodes and thresholds as a function of prior state, therapist action, and context. This could yield testable mechanistic hypotheses, such as whether particular action classes increase the probability of PRO under B-level thresholds, or whether “protect positives” actions reduce the probability of SUP following PRO in vulnerable patients.

A key advantage of coupling PAD-S with equation discovery is interpretability. Rather than treating the therapy session as a black-box sequence, PAD-S constrains the state space to clinically meaningful variables and safety gating, which can reduce the risk of overfitting and improve cross-setting transportability. **Figure 1B** summarizes this interface from transcript episodes to structured datasets and hybrid modeling outputs.

## 5 Research agenda: testable hypotheses and feasible pilot designs

PAD-S is intended as a hypothesis-generating and hypothesis-testing bridge between clinical process knowledge and computational models. Below, I outline example hypotheses and practical pilot designs that can be executed with modest resources and that directly address the feasibility, reliability, and scientific value of PAD-S annotation.

### 5.1 Example testable hypotheses

1. H1 (policy safety adherence): Across therapists, C-level threshold markers will be followed predominantly by safeguarding actions (regulation/repair). Higher safety adherence (fewer deepening actions at C) will be associated with fewer alliance ruptures and lower dropout.
2. H2 (dose–tolerance matching): Therapists will preferentially use graded dosing under B-level thresholds and standard dosing under A-level thresholds. Greater dose–tolerance matching will predict functional gains over treatment.
3. H3 (protect-positives mechanism): In patients with prominent self-criticism/shame, PRO episodes will be followed by SUP more often unless the therapist implements “protect positives” actions. Protect-positives actions will reduce the probability of PRO → SUP transitions.
4. H4 (model discovery and responder signatures): Applying equation discovery (e.g., SPICE) to PAD-S trajectories will yield sparse transition equations. Responders vs. nonresponders will show distinct structural patterns (e.g., stronger DEF attractors, higher SUP reactivity, or reduced sensitivity to therapist actions) that can be tested prospectively.

### 5.2 Feasible pilot study designs

The following pilots are structured to (i) establish annotation reliability, (ii) link PAD-S process metrics to functional outcomes, and (iii) test the value of hybrid modeling on PAD-S-labeled data.

Pilot 1: Annotation feasibility and interrater reliability.

- Sample: 10–20 de-identified session transcripts (diverse modalities and case mixes).
- Raters: 3–5 trained raters; iterative calibration with a shared rater pack.
- Unit: episode line segmentation at clinically meaningful decision points (rather than continuous second-by-second coding).
- Outcomes: interrater reliability for node (DEF/ANX/PRO/SUP) and threshold (A/B/C). Target: κ ≥ 0.70 for node and threshold; ICC for any continuous ratings if used.
- Deliverable: refined codebook and adjudication rules; estimate of annotation time per session.

Pilot 2: Functional linkage study.

- Sample: ~30–60 patients with baseline and follow-up functional assessment (e.g., Mini-ICF-APP).
- Measures: derive PAD-S process metrics per case (e.g., proportion of PRO time, frequency of C markers, PRO → SUP transitions, dose–tolerance matching index).
- Hypothesis test: regress functional change on PAD-S metrics while controlling for baseline severity; focus on effect sizes and feasibility rather than definitive inference.

Pilot 3: Hybrid modeling/equation discovery on PAD-S trajectories.

- Data: PAD-S episode-line trajectories from Pilot 2 (or larger corpus if available).
- Modeling: fit a sequence model to capture dynamics; apply sparse regression/equation discovery (e.g., SPICE) to recover interpretable transition equations.
- Evaluation: predictive generalization (held-out sessions/cases), stability of discovered equations across bootstraps, and clinical interpretability (expert review).
- Deliverable: candidate mechanistic equations and responder/nonresponder signatures for prospective testing.

### 5.3 Feasibility demonstration: proof-of-concept episode-line annotation

Feasibility benefits from a concrete example. Below is a short proof-of-concept that illustrates how PAD-S translates a brief therapist–patient exchange into one episode line and a minimal JSON record. The excerpt is a fictional composite created for training and illustration; it does not reproduce copyrighted or identifiable clinical material.

Therapist: “When your manager criticized you, what happened inside—right now as you remember it?”

Patient: “My stomach clenched and I went blank. I told him it’s fine.”

Episode line (human-readable): Trigger=manager criticism → Node=ANX (with DEF detour) → Threshold=B (narrowing; smooth-muscle activation) → Action=downshift + grounding + graded micro-focus → Functional target=endurance/persistence & planning/structuring → Re-check=4–6 weeks.

JSON example: {“trigger”:”manager criticism”,”node”:”ANX”,”secondary_node”:”DEF”,”threshold”:”B”,”action”:[“grounding”,”graded_focus”],”function_target”:[“endurance”,”planning”],”recheck_weeks”:6}.

## 6 Discussion

PAD-S is proposed as a compact, safety-gated state–action grammar for psychotherapy micro-decisions. It translates pragmatic process heuristics from experiential dynamic psychotherapy into a form that can be (i) annotated in transcripts, (ii) logged in clinical documentation as episode lines, and (iii) used as an interface to hybrid modeling approaches such as equation discovery. The framework is intentionally minimal: it aims to capture a decision layer (node, threshold, action) that can be combined with richer descriptive coding systems when research questions require finer granularity.

Several limitations are important. First, PAD-S is grounded in a specific clinical tradition (EDT/ISTDP) and therefore inherits its assumptions about what cues and interventions matter. The translation stance described here is a pragmatic proposal, not an empirical claim of equivalence between modalities. Second, PAD-S is not a substitute for clinical judgment; it formalizes heuristics and safety gating but cannot capture the full nuance of case formulation, ethics, or relational context.

Feasibility and reliability are central. Episode-line coding reduces cognitive load compared with continuous micro-coding, but it still requires training, calibration, and transparent adjudication rules. Supplementary rater materials can support a calibration workflow in which raters first converge on segmentation rules and then on node/threshold assignments. In practice, reliability will likely vary by node (e.g., PRO and SUP may require more context) and by setting. Reporting annotation time, κ/ICC, and failure modes should be treated as primary outcomes in early studies.

Finally, governance and safety must remain explicit. PAD-S should be used to support human learning (training, supervision, hypothesis testing), not to automate treatment decisions. If machine learning is used for pre-annotation or decision support, therapists must retain human-final control, and privacy-preserving workflows are required (19).

## Data Availability

The original contributions presented in the study are included in the article/**Supplementary Material**. Further inquiries can be directed to the corresponding author/s.
