# Supplementary material for: Perceive–Assess–Dose–Safeguard: a safety-gated state–action grammar for psychotherapy micro-decisions in computational psychiatry
Source: Front Psychiatry. 2026 Mar 13;17:1749364. doi: 10.3389/fpsyt.2026.1749364 (PMC13023058; doi:10.3389/fpsyt.2026.1749364)
Supplement: Supplementary file 1 [file DataSheet1.pdf]

Supplementary Material S1 – PAD-S Codebook and Decision Matrix

Corresponding author: Dr. med. Eik Niederlohmann – Kliniken Erlabrunn, Department of Psychosomatic Medicine and Psychotherapy, Breitenbrunn, Germany – kontakt@praxis-niederlohmann.de

Legend

This supplement introduces the PAD-S codebook and decision matrix. It defines nodes, thresholds, Node×Threshold rules, pseudocode, and data fields for constructing PAD-S episode lines. PAD-S is theoretically grounded in experiential dynamic psychotherapy (EDT/ISTDP) but expressed as an orientation-translatable representation layer for computational use (e.g., DEF as avoidance/safety behavior; ANX as arousal/tolerance; PRO as approach and emotional access; SUP as self-criticism/shame). PAD-S grew out of an earlier clinician-facing conflict-square formulation (CSA) (Niederlohmann, 2025) and is refined here as an explicit state–action representation optimized for annotation and modeling.

1 Core Constructs

1.1 Front-of-system nodes

Node	Label	Definition and observable markers
DEF	Defense	Cognitive/behavioral maneuvers that avoid affect or relationship (generalization, joking, intellectualizing, monologuing, debating).
ANX	Anxiety / Affect tolerance	Striated tension (jaw/shoulders), smooth-muscle markers (GI flip, nausea), cognitive–perceptual disruption (fogging, spacing out).
PRO	Progress / Feeling	Naming a wish/need, regulated affect expression, agency, responsive eye contact, accepting help.
SUP	Superego / Shame	Global self-attack, contempt, collapse after positive emotion (joy→attack→collapse).

1.2 Thresholds (A/B/C) – window of tolerance

Threshold	Label	Clinical description
A	Regulated	Affect is symbolizable; standard dosing possible.
B	Narrowing	Window is narrowing; graded dosing required.
C	Collapse	CPD or shame collapse; stop deepening and regulate.

2 Node × Threshold → Next Action (Decision Matrix)

Node	A (regulated)	B (narrowing)	B→C (drift)	C (collapse)
DEF	Gentle block + link to cost	Partial block, keep focus small	Stop challenge, regulate	No confrontation; co-regulate
ANX	Name pathway; short exposure	Graded exposure + breath	Prioritize regulation	Stop, orient, regulate
PRO	Validate + link to action	Reinforce micro-steps	Protect positives	Pause; rebuild safety
SUP	Reality-test attack	Protect positives; name pattern	Stop-rule: protect	Shame repair; no deepening

3 PAD-S Episode Line: Pseudocode

(for illustration only, not a normative standard)

```
def pad_s_step(event):
    # event contains: trigger, patient_utterance, nonverbal, physiology

    signals = perceive_signals(event)
    node = classify_node(signals)          # DEF | ANX | PRO | SUP
    threshold = classify_threshold(signals) # A | B | C (or B->C)

    if threshold == 'C':
        actions = ['stop_deepening', 'regulate', 'protect_positives']
    else:
        actions = lookup_actions(node, threshold)
        if threshold == 'B->C':
            actions.insert(0, 'switch_to_graded')

    episode_line = {
        'episode_id': make_id(),
        'trigger': event.get('trigger'),
        'patient_utterance': event.get('patient_utterance'),
        'node': node,
        'threshold': threshold,
        'actions': actions,
        'mini_icf_targets': infer_targets(node, threshold)
    }
    log_episode(episode_line)
    return episode_line
```

4 Data Dictionary for PAD-S Episode Lines

Field	Type	Description
episode_id	string	Unique ID (session + sequence).
session_id	string	Session identifier.
timecode	string	Approx. timestamp (e.g., “00:12:34”).
trigger	string	Clinician prompt or situational cue.
patient_utterance	string	De-identified patient response.
node	string	DEF, ANX, PRO, SUP.
threshold	string	A, B, C (optional B→C).
actions	list	Therapist interventions.
mini_icf_targets	list	Mini-ICF-APP domains.
annotator_id	string	Rater code.
