# Supplementary material for: Perceive–Assess–Dose–Safeguard: a safety-gated state–action grammar for psychotherapy micro-decisions in computational psychiatry
Source: Front Psychiatry. 2026 Mar 13;17:1749364. doi: 10.3389/fpsyt.2026.1749364 (PMC13023058; doi:10.3389/fpsyt.2026.1749364)
Supplement: Supplementary file 2 [file DataSheet2.pdf]

Supplementary Material S2 – PAD-S Annotated Transcripts and Rater Pack

Corresponding author: Dr. med. Eik Niederlohmann – Kliniken Erlabrunn, Department of Psychosomatic Medicine and Psychotherapy, Breitenbrunn, Germany – kontakt@praxis-niederlohmann.de

Legend

This supplement provides de-identified PAD-S-annotated transcript excerpts and minimal rater guidance. It illustrates how PAD-S episode lines are derived from clinical dialogue and supports training, supervision, reliability (κ/ICC), and model development.

Note: All transcript excerpts below are fictionalized composites created for training and illustration; no identifiable clinical data are included.

1 Coding Legend

- PAD-S episode line: Trigger → Response → Threshold (A/B/C) → Action → Mini-ICF-APP impact
- Nodes: DEF / ANX / PRO / SUP Thresholds: A (regulated), B (narrowing), C (collapse)

2 JSON Schema (Minimal Example)


```
{
 "episode_id": "sess01_e05",
 "session_id": "sess01",
 "timecode": "00:07:32",
 "trigger": "When your manager raised his voice, what went through you?",
 "patient_utterance": "My stomach knotted and I said it's fine.",
 "node": "ANX",
 "threshold": "B",
 "actions": ["graded_exposure", "breath_pacing"],
 "mini_icf_targets": ["endurance", "planning"],
 "annotator_id": "R01",
 "notes": "smooth-muscle anxiety; brief drift toward C avoided"
}
```


3 Rater Training Steps

1. Study PAD-S (S1).
2. Joint annotation of 10–15 clips with expert guide.
3. Independent coding of calibration set (n≈20 episodes).
4. Aim for node $\kappa \geq 0.70$ and threshold $\kappa \geq 0.65$ (use ICC for any continuous ratings).

4 Example Transcript A – Attachment Cue & SUP Whiplash

(All transcript excerpts are fictionalized composites based on routine ISTDP/EDT practice and have been de-identified; no single patient can be re-identified.)

t	Spk	Utterance (de-identified)	Node	Th	Action	Notes
00:00	Th	"When your manager frowned … what went through you?"	–	–	Trigger	Authority cue
00:06	Pt	"My chest tightened; I thought I messed up."	ANX	B	Name anxiety	Striated tension
00:14	Th	"Stay with that tightness for 2 seconds…"	ANX	B	Graded exposure	2–3 s → regulate
00:26	Pt	"I wanted him to see I tried."	PRO	A	Validate + link to need	Emerging wish
00:36	Pt	"That's silly… should toughen up."	SUP	B	Protect positives	Joy→attack
00:46	Th	Protecting the part that wanted connection	SUP	B	Safeguard	Avoid SUP collapse

5 Example Transcript B – Smooth-Muscle Anxiety

(All transcript excerpts are fictionalized composites based on routine ISTDP/EDT practice and have been de-identified; no single patient can be re-identified.)

t	Spk	Utterance	Node	Th	Action	Notes
00:00	Th	"Let's return to the moment…"	–	–	Trigger	Relationship cue
00:08	Pt	"Stomach flipped; changed topic."	ANX	B	Name smooth-muscle ANX	GI flip
00:15	Th	"Notice flip for 2 s…"	ANX	B	Micro-exposure + regulate	Safety focus
00:25	Pt	Catastrophic thought	DEF	A	Clarify defense	Cognitive detour
00:36	Th	Invite wish/need	PRO	A	PRO step	Low dose
00:48	Pt	"Hoped they wouldn't leave me."	PRO	A	Validate + Mini-ICF	Dyadic relatedness

6 Clip-Level Scoring Sheet (template)

Use one row per coded clip/episode. Duplicate rows as needed.

Clip ID	Dominant node	Peak threshold	Dose adherence (0–3)	Safety adherence (0–3)	Mini-ICF notes
