# Supplementary material for: Perceive–Assess–Dose–Safeguard: a safety-gated state–action grammar for psychotherapy micro-decisions in computational psychiatry
Source: Front Psychiatry. 2026 Mar 13;17:1749364. doi: 10.3389/fpsyt.2026.1749364 (PMC13023058; doi:10.3389/fpsyt.2026.1749364)
Supplement: Supplementary file 3 [file DataSheet3.pdf]

Supplementary Material S3 – PAD-S to Mini-ICF-APP Crosswalk and Phrase Bank

Corresponding author: Dr. med. Eik Niederlohmann – Kliniken Erlabrunn, Department of Psychosomatic Medicine and Psychotherapy, Breitenbrunn, Germany – kontakt@praxis-niederlohmann.de

Legend

This supplement maps PAD-S episode patterns to Mini-ICF-APP functional domains and provides copy-ready phrasing for clinical documentation and computational outcome construction.

(The phrases are intended as examples; clinicians should adapt wording to local documentation standards and patient preferences.)

1 Crosswalk Examples

PAD-S Pattern	Typical Trigger	Mini-ICF Domains	Functional Phrasing	Targets & Re-Check
ANX B→C (attachment)	Partner criticism, authority	Endurance, Planning, Dyadic relatedness	“Under attachment-laden triggers endurance and planning drop quickly; closeness oscillates. Graded exposure + regulation required.”	3 micro-asks/week; 2 graded exposures/session; 6–8 weeks
DEF@A/B + PRO	Performance feedback	Decision-making, Planning	“In stress you briefly avoid decisions but re-engage with support; planning improves with structured practice.”	1 structured decision/week; 4–6 weeks
SUP after PRO (joy→attack)	Praise, success	Positive emotion, Interpersonal interactions	“After successes you attack/minimize yourself, limiting enjoyment; we aim to protect positives.”	Positive review each session; 1 shared success/week; 4–6 weeks

2 Phrase Bank (by Mini-ICF Domain)

Mini-ICF Domain	Example phrasing (editable template)
Endurance	“Under emotional stress your endurance drops; we will use short, repeated steps to build stamina.”
Planning/Structuring	“When anxiety rises you lose structure; breaking tasks into 2–3 parts with cues should help.”
Decision-Making	“Self-attack slows decisions; we will practice time-limited choices.”
Assertiveness	“Fear of conflict and inner attack reduce assertiveness; we practice one value-congruent request/week.”
Dyadic Relatedness	“Contact oscillates between approach and withdrawal; graded contact stabilizes closeness.”
Group Interaction	“You withdraw in groups when shame/anxiety rise; predictable roles will support participation.”
